# Association between the atherogenic index of plasma and new-onset non-alcoholic fatty liver disease in non-obese participants

**DOI:** 10.3389/fendo.2022.969783

**Published:** 2022-08-18

**Authors:** Kemin Li, Ji Li, Xiaoyun Cheng, Jing Wang, Jingnan Li

**Affiliations:** ^1^ Department of Gastroenterology, Peking Union Medical College Hospital, Peking Union Medical College, Chinese Academy of Medical Sciences, Beijing, China; ^2^ Key Laboratory of Gut Microbiota Translational Medicine Research, Peking Union Medical College Hospital, Peking Union Medical College, Chinese Academy of Medical Sciences, Beijing, China

**Keywords:** atherogenic index of plasma, non-alcoholic fatty liver disease, non-obese participants, longitudinal association, obesity

## Abstract

**Introduction:**

Non-alcoholic fatty liver disease (NAFLD) in the non-obese population accounts for a large proportion of NAFLD. Atherogenic index of plasma (AIP, defined as the logarithm of the triglyceride/high-density lipoprotein cholesterol ratio.) can provide a stronger reflection of dyslipidemia and studies on the longitudinal association between AIP and NAFLD were limited in non-obese participants, especially in different BMI groups.

**Methods:**

We performed a *post-hoc* analysis of data obtained from the Dryad data repository *(Dryad is a nonprofit open database of medicine.)* and explored the predictive value of AIP on the risk of NAFLD among non-obese participants.

**Results:**

This study included 16173 participants with AIP, of which 2322(14.4%) non-obese participants developed into individuals with NAFLD with the 5-year follow-up examination. The difference between AIP quartiles in the cumulative estimation of new-onset NAFLD was significant, and with increased AIP, the cumulative new-onset NAFLD gradually increased. Participants in higher AIP quartiles had a significantly increased risk of NAFLD. In the fully adjusted model 3, hazard ratios of the new-onset NAFLD for subjects in Q2, Q3, and Q4 of AIP were 2.00 (1.59, 2.53), 2.61 (2.09, 3.72), and 4.49 (3.62, 5.57) respectively. Meanwhile, the trend test for the association between AIP quartiles and the new-onset NAFLD presented that AIP quartile was positively and strongly associated with the new-onset NAFLD (adjusted hazard ratio (95%CI) in Model 3: 1.59 (1.51, 1.67), *P*<0.001). We found that AIP was also positively and strongly associated with new-onset NAFLD in different sex groups and different age groups in female patients. Moreover, the predictive ability of AIP was no significant difference in different sex groups and different age groups in female patients. In the subgroup analysis, we found that in the low BMI population, the predictive effect of AIP for new-onset NAFLD was expanded by 2-3 times for each quality increase of AIP.

**Conclusion:**

This study found that AIP was a strong independent risk factor for new-onset NAFLD among non-obese individuals especially in the low BMI participants, and screening for AIP in this population can be used to prevent future NAFLD.

## Introduction

Non-alcoholic fatty liver disease (NAFLD) is a chronic liver disease where hepatic fat accumulation in the absence of quantities of alcohol and any other secondary cause, diagnosed by pathology or imaging (1). Nowadays, NAFLD is gradually becoming the leading cause of chronic liver disease, the prevalence is estimated to be 25% in the worldwide (2), and ranges from 15% to 40% in Asia (3). The pathogenesis of NAFLD was not clearly, however, steatosis was a key factor in its development (4).

Atherogenic index of plasma (AIP), calculated by the logarithm of the ratio between the level of triglyceride and high-density lipoprotein cholesterol (TG/HDL-C), is an indicator reflecting the characteristics and the degree of abnormal lipid metabolism (5),and previous researches found AIP was associated with NAFLD (6–8). However, most of the previous studies were cross-sectional, did not explain the longitudinal association between AIP and NAFLD.

It is wide known that obesity was strongly associated with NAFLD, but the non-obese population is still a very large population suffering from NAFLD. It was reported that the rates of NAFLD in non-obese population average 10%-30% in Western and Eastern countries (9), and as high as 17.5% in China (10). Moreover, non-obese individuals with NAFLD had a significantly higher cardiovascular disease (CVD) risk compared with obese subjects with NAFLD (11). However, previous studies did not focus on non-obese people, and relevant studies on NAFLD prediction were limited, and no appropriate evaluation index was found.

It is crucial to concentrate on NAFLD in the non-obese population, and a good indicator is needed to predict the occurrence of NAFLD. Therefore, our study evaluated the relationship between AIP and new-onset NAFLD in non-obese population, aiming to explore the predictive value of AIP on the risk of non-obese patients with NAFLD.

## Methods

Data we used in this study was derived from the Dryad data repository at http://datadryad.org/ with the doi: 10.5061/dryad.1n6c4.

### Study population and design

The participants in the longitudinal studies were individuals who received a health examination in Wenzhou Medical Center of Wenzhou People’s Hospital from January 2010 to December 2014. The protocol and main outcomes were previously published (11).The major outcomes were that increased normal low-density lipoprotein cholesterol (LDL-C) levels were related to an elevated risk of NAFLD (diagnosed by the ultrasound and accompanied with alcohol consumption (≤140g/week for men and ≤70g/week for women)) in non-obese (BMI<25kg/m^2^) populations independently. And this study was approved by the ethics committee of Wenzhou People’s Hospital.

Our aim of this analysis was to assess the association between baseline AIP (calculated by TG and HDL-C) and the new-onset NAFLD outcomes in non-obese individuals. And 16371 participants were included in the final analysis.

### Evaluation of atherogenic index of plasma and study outcomes

The AIP was calculated by the logarithm of TG/HDL-C mole ratio base 10. In total, 16173participants were grouped according to AIP quartiles in ascending order: Q1 (n=4043), Q2 (n=4043), Q3 (n=4043), Q4 (n=4044) and the Q1 group was used as the reference. New-onset NAFLD was the outcome of this analysis. The definition of the outcomes was published in the Sun et al. study protocol (12).

### Statistical analysis

Included participants were grouped by the AIP quartiles. Continuous variables were expressed as mean (standard deviation) or median (Q1-Q3) based on the distribution of data. The difference between the quartiles were tested using ANOVA and Kruskal-Wallis H test for normal distribution data and skew distribution data respectively. Chi-square test or Fisher test were applied to compare the categorical variables. All categorical variables were expressed as frequency (percentile).

The Kaplan-Meier analysis with log-rank test was used to estimate cumulative incidence of new-onset NAFLD in non-obese populations and to compare in the AIP quartiles.

The Cox model was applied to evaluate the association between AIP quartiles and the occurrence of the new-onset NAFLD in three models. Model 1 was not adjusted. Model 2 was adjusted for age, sex and body mass index (BMI). Model 3 was adjusted for age, sex, BMI, systolic blood pressure (SBP), diastolic blood pressure (DBP), alkaline phosphatase (ALP), Gamma glutamyl transferase (GGT), alanine aminotransferase (ALT), aspartate aminotransferase (AST), serum creatinine (CR), and uric acid (UA). We performed Schoenfeld residuals test to test the proportional hazard assumption in the Cox model. The relationship between AIP and new-onset NAFLD according to various subgroups were assessed with stratified analysis and interaction test by using Model 3.

All analyses were performed using the statistical software packages R (The R Foundation; http://www.R-project.org). Statistical significance was set at a two-tailed *P*<0.05.

## Results

### Baseline characteristics of the participants

In total,16173 participants with AIP were included in this analysis, and 2322(14.4%) non-obese participants developed into individuals with NAFLD with the 5-year follow-up examination. The baseline characteristics of the included participants according to the AIP quartiles were shown in **Table 1**. Participants in higher quartiles of AIP tended to be male, elder and were more likely to have a higher BMI, SBP, DBP liver enzymes (including ALP, GGT, ALT, AST), CR, UA, blood lipids (including TC, TG, HDL-C, LDL-C), fasting triglycerides, glucose, and NAFLD risk than in lower quartiles.

**Table 1:** Baseline characteristic of the participants included in the analysis according to AIP quartiles.

Variables	AIP				P value
	Q1	Q2	Q3	Q4	
N	4043	4043	4043	4044	
AIP, mean ± SD	-0.42 ± 0.10	-0.21 ± 0.05	-0.03 ± 0.06	0.27 ± 0.18	<0.001
Female, n (%)	2094 (51.79%)	1993 (49.30%)	1877 (46.43%)	1726 (42.68%)	<0.001
BMI (Kg/m^2^), mean ± SD	20.46 ± 1.94	21.06 ± 2.00	21.58 ± 1.97	22.43 ± 1.75	<0.001
Age, mean ± SD	42.43 ± 14.72	42.66 ± 14.83	43.57 ± 14.98	44.25 ± 15.24	<0.001
SBP (mm Hg), mean ± SD	115.64 ± 15.77	118.95 ± 16.45	122.06 ± 16.35	126.26 ± 16.43	<0.001
DBP (mm Hg), mean ± SD	69.71 ± 9.61	71.73 ± 9.95	73.39 ± 10.23	76.40 ± 10.43	<0.001
ALT(U/L), median (Q1-Q3)	14.00 (11.00-18.00)	15.00 (12.00-21.00)	17.00 (13.00-23.00)	20.00 (15.00-28.00)	<0.001
AST(U/L), median (Q1-Q3)	20.00 (17.00-24.00)	21.00 (18.00-25.00)	21.00 (18.00-25.00)	23.00 (20.00-27.00)	<0.001
ALP(U/L), mean ± SD	64.98 ± 20.71	70.39 ± 25.11	74.21 ± 23.67	77.90 ± 21.21	<0.001
GGT(U/L), median (Q1-Q3)	17.00 (14.00-22.00)	19.00 (15.00-26.00)	22.00 (17.00-32.00)	29.00 (21.00-44.50)	<0.001
CR (umol/L), median (Q1-Q3)	69.00 (61.00-79.00)	75.00 (63.00-88.00)	79.00 (66.00-93.00)	84.00 (71.00-96.00)	<0.001
UA (umol/L), median (Q1-Q3)	227.00 (189.00-276.00)	250.00 (205.00-310.00)	285.00 (232.00-342.00)	325.00 (267.00-384.00)	<0.001
TC (mmol/L), mean ± SD	4.57 ± 0.74	4.56 ± 0.72	4.62 ± 0.72	4.75 ± 0.78	<0.001
TG (mmol/L), median (Q1-Q3)	0.68 (0.58-0.78)	0.94 (0.83-1.07)	1.24 (1.09-1.42)	1.98 (1.63-2.53)	<0.001
HDL-C (mmol/L), mean ± SD	1.81 ± 0.33	1.54 ± 0.28	1.36 ± 0.25	1.14 ± 0.21	<0.001
LDL-C (mmol/L), mean ± SD	2.11 ± 0.46	2.23 ± 0.46	2.34 ± 0.45	2.37 ± 0.45	<0.001
Fasting glucose (mmol/L), mean ± SD	4.99 ± 0.63	5.10 ± 0.73	5.17 ± 0.78	5.32 ± 0.92	<0.001
NAFLD	117 (2.89%)	315 (7.79%)	550 (13.60%)	1340 (33.14%)	<0.001

AIP, atherogenic index of plasma; BMI, body mass index (BMI);SBP, systolic blood pressure; DBP, diastolic blood pressure; ALP, alkaline phosphatase; GGT, gamma glutamyl transferase; ALT, alanine aminotransferase; AST, aspartate aminotransferase; CR, serum creatinine; UA, uric acid; TC, total cholesterol; TG, total triglycerides; HDL-C, high-density lipoprotein cholesterol; LDL-C, low-density lipoprotein cholesterol.

### Kaplan-Meier curves analysis

**Figure 1:**
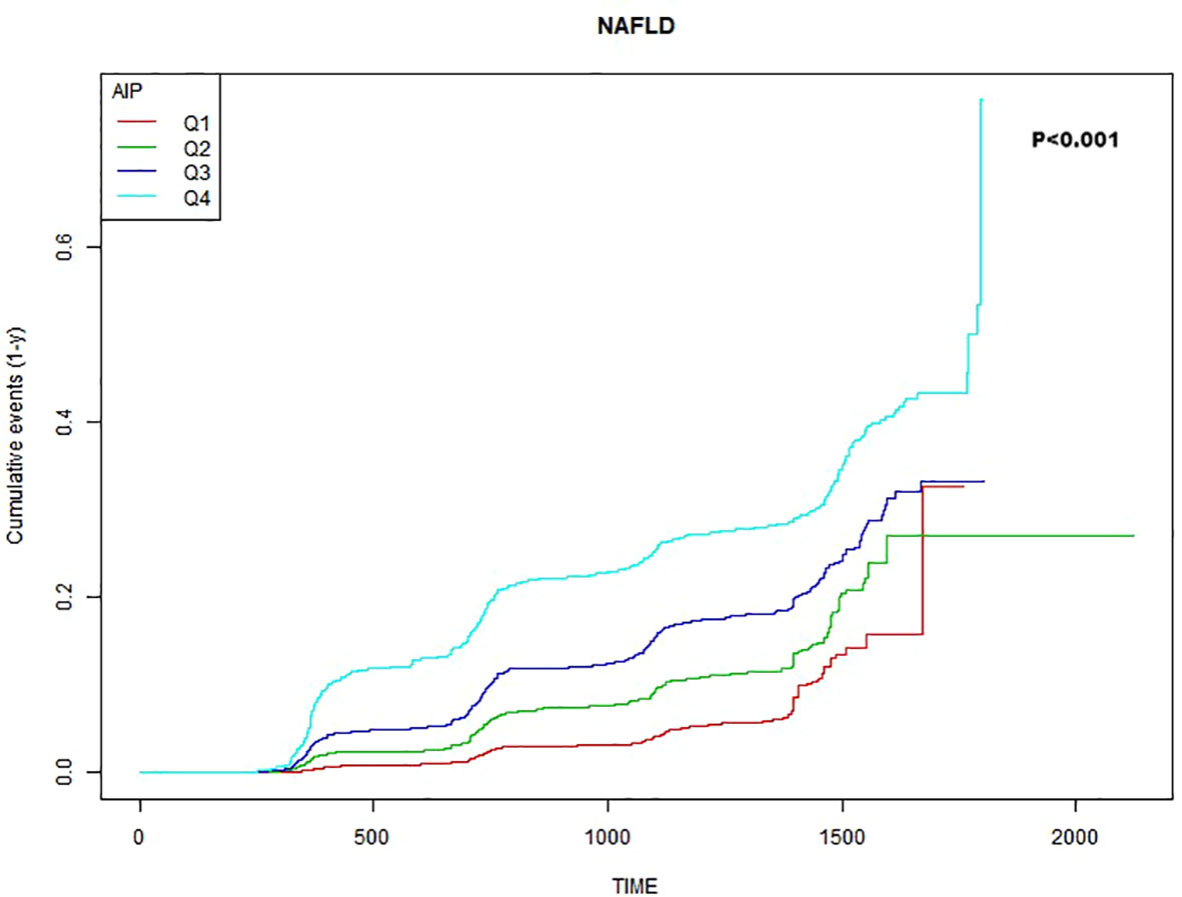
Kaplan–Meier estimation of new-onset NAFLD by AIP quartiles.

**Figure 1** showed the Kaplan-Meier curves of the cumulative incidence of new-onset NAFLD stratified by AIP quartiles. NAFLD incident risk was significantly different between each level of AIP(*P*<0.001). With increased AIP, the cumulative new-onset NAFLD gradually increased. And the top quartile group performed the maximum risk of NAFLD with no doubt.

### Relationship between AIP and new-onset NAFLD

**Table 2** exhibited the hazard ratios (HRs) (95%CI) of new-onset NAFLD among the included subjects grouped by quartiles of AIP. After adjustment for potential confounding factors, including age, sex, BMI, SBP, DBP, ALP, GGT, ALT, AST, CR, UA, the associations remained significant (*P*< 0.001). Especially in the fully adjusted Model 3, the adjusted HRs of the new-onset NAFLD for subjects in Q2, Q3, and Q4 of AIP were 2.00 (1.59, 2.53), 2.61 (2.09, 3.72), and 4.49 (3.62, 5.57) respectively. Meanwhile, the trend test for the association between AIP quartiles and the new-onset NAFLD presented that AIP quartile was positively and strongly associated with the new-onset NAFLD (adjusted HR (95%CI) in Model 3: 1.59 (1.51, 1.67), *P*<0.001).

**Table 2:** Association between AIP and new-onset NAFLD in different models.

AIP quartiles	Model 1	Model 2	Model 3
	HR (95%CI) P value		
1	Ref.	Ref.	Ref.
2	2.70 (2.18, 3.34) P<0.001	2.09 (1.69, 2.59) P<0.001	2.00 (1.59, 2.53) P<0.001
3	4.57 (3.75, 5.59) P<0.001	2.93 (2.40, 3.58) P<0.001	2.61 (2.09, 3.27) P<0.001
4	11.72 (9.70, 14.16) P<0.001	5.54 (4.57, 6.71) P<0.001	4.49 (3.62, 5.57) P<0.001
P for trend (1 Q increment)	2.22 (2.12, 2.32) P<0.001	1.72 (1.64, 1.80) P<0.001	1.59 (1.51, 1.67) P<0.001

Model 1 was not adjusted. Model 2 was adjusted for age, sex and BMI. Model 3 was adjusted for age, sex, BMI, SBP, DBP, ALP, GGT, ALT, AST, CR, UA.

**Supplemental Table 1** exhibited the hazard ratios (HRs) (95%CI) of new-onset NAFLD among the included subjects grouped by quartiles of TG and HDL-C, respectively. Both variables were associated with new-onset NAFLD after adjustment for potential confounding factors (age, sex, BMI, SBP, DBP, ALP, GGT, ALT, AST, CR, UA).

### Relationship between AIP and new-onset NAFLD according to sex

We further analyzed this relationship between AIP and new-onset NAFLD according to sex as **Table 3** showed. We found that no matter male and female, AIP was also strongly associated with the new-onset NAFLD. Specifically, after adjusting for potential factors (age, BMI, SBP, DBP, ALP, GGT, ALT, AST, CR and UA), the association was still significant (P< 0.001) even in fully adjusted Model 3. The trend test showed that AIP quartile was positively and significantly associated with new-onset NAFLD (Male: HR (95%CI) in Model 3: 1.60 (1.48, 1.73), P<0.001; Female: HR (95%CI) in Model 3: 1.57 (1.46, 1.68), P<0.001). However, the interaction of sex and AIP had no significant effect on the risk of NAFLD (P for interaction >0.05).

**Table 3:** Association between AIP and new-onset NAFLD according to sex.

	Model 1	Model 2	Model 3
	HR (95%CI) P value		
Sex= male AIP quartiles			
Q1	Ref.	Ref.	Ref.
Q2	2.87 (2.11, 3.90) P<0.001	2.26 (1.66, 3.08) P<0.001	2.36 (1.66, 3.37) P<0.001
Q3	4.92 (3.69, 6.57) P<0.001	3.13 (2.34, 4.18) P<0.001	2.97 (2.12, 4.16) P<0.001
Q4	12.38 (9.41, 16.30) P<0.001	5.89 (4.45, 7.78) P<0.001	5.07 (3.64, 7.05) P<0.001
P for trend (1 Q increment)	2.23 (2.08, 2.39) P<0.001	1.73 (1.61, 1.85) P<0.001	1.60 (1.48, 1.73) P<0.001
Sex= female AIP quartiles			
Q1	Ref.	Ref.	Ref.
Q2	2.53 (1.89, 3.40) P<0.001	1.94 (1.44, 2.60) P<0.001	1.74 (1.28, 2.38) P<0.001
Q3	4.24 (3.21, 5.58) P<0.001	2.74 (2.08, 3.61) P<0.001	2.29 (1.70, 3.07) P<0.001
Q4	10.97 (8.45, 14.24) P<0.001	5.22 (4.01, 6.80) P<0.001	3.96 (2.98, 5.26) P<0.001
P for trend (1 Q increment)	2.20 (2.06, 2.34) P<0.001	1.71 (1.61, 1.83) P<0.001	1.57 (1.46, 1.68) P<0.001
P for interaction	0.905	0.912	0.604

Model 1 was not adjusted. Model 2 was adjusted for age and BMI. Model 3 was adjusted for age, BMI, SBP, DBP, ALP, GGT, ALT, AST, CR, UA.

We also proceeded the analysis of the association between AIP and new-onset NAFLD in different age groups in female patients. We considered age of >=50 as a surrogate for menopausal status. As shown in **Table 4**, in the female group of age<50, especially in fully adjusted Model 3, the adjusted HRs of the new-onset NAFLD for subjects in Q2, Q3, and Q4 of AIP were 1.93 (1.24, 3.00), 2.66 (1.75, 4.04), and 1.68 (1.53, 1.84). While in the female patients over 50 years old, the adjusted HRs of the new-onset NAFLD for subjects in Q2, Q3, and Q4 of AIP were 1.64 (1.05, 2.57), 2.04 (1.34, 3.12), and 1.42(1.28, 1.58) respectively. Moreover, the interaction test revealed that we found a significant interaction between age (<50 vs. ≥50, P for interaction <0.05) and AIP on the risk of NAFLD in female patients in Model 1. However, in the adjusted Model (Model2 and Model3), AIP had no significant effect on the risk of NAFLD between the different age groups (P values of interaction were all >0.05) but showed a trend of weaker predictive capabilities in menopausal female patients.

**Table 4:** Association between AIP and new-onset NAFLD in different age groups in female patients.

	Model 1	Model 2	Model 3
	HR (95%CI) P value		
Age <50 years AIP quartiles			
Q1	Ref.	Ref.	Ref.
Q2	3.07 (2.02, 4.68) P<0.001	2.36 (1.55, 3.60) P<0.001	1.93 (1.24, 3.00) P=0.003
Q3	5.69 (3.82, 8.48) P<0.001	3.48 (2.33, 5.19) P<0.001	2.66 (1.75, 4.04) P<0.001
Q4	15.25 (10.42, 22.32) P<0.001	7.00 (4.76, 10.28) P<0.001	4.97 (3.32, 7.44) P<0.001
P for trend (1 Q increment)	2.39 (2.19, 2.60) P<0.001	1.83 (1.68, 2.00) P<0.001	1.68 (1.53, 1.84) P<0.001
Age ≥50 years AIP quartiles			
Q1	Ref.	Ref.	Ref.
Q2	2.15 (1.42, 3.25) P<0.001	1.61 (1.06, 2.44) P=0.026	1.64 (1.05, 2.57) P=0.029
Q3	3.01 (2.04, 4.44) P<0.001	2.13 (1.44, 3.15) P<0.001	2.04 (1.34, 3.12) P<0.001
Q4	7.36 (5.13, 10.55) P<0.001	3.74 (2.59, 5.38) P<0.001	3.08 (2.06, 4.60) P<0.001
P for trend (1 Q increment)	1.95 (1.77, 2.15) P<0.001	1.56 (1.41, 1.71) P<0.001	1.42 (1.28, 1.58) P<0.001
P for interaction	0.012	0.065	0.081

Model 1 was not adjusted. Model 2 was adjusted for BMI. Model 3 was adjusted for BMI, SBP, DBP, ALP, GGT, ALT, AST, CR, UA.

### Subgroup analysis for the risk of new-onset NAFLD by baseline AIP quartiles

We further explored other risks in the associations between AIP (per quartile increment) and new-onset NAFLD by performing a subgroup analysis to estimate the factors that might influence the results. As shown in **Table 5**, we used sex (male vs. female), age (<40 years vs. ≥40 years), BMI (<20.4 vs. 20.4 to ≤22.5 vs. ≥22.5), ALT (16U/L vs. ≥16 U/L) and SBP (<120mmHg vs. ≥120mmHg) as the prespecified subgroups. We found a significant interaction between BMI (<20.4 vs. ≥20.4 to ≤22.5 vs. ≥22.5, P for interaction <0.001) and AIP on the risk of NAFLD. The effect of AIP on the risk of NAFLD showed stronger predictive capabilities in lower BMI. The HRs of the effect of AIP on the risk of NAFLD in BMI tertiles were 2.32(1.86,2.90), 1.84(1.66,2.03),1.48(1.40,1.57) from the bottom to the top. In addition, there was also a significant interaction between SBP (<120mmHg vs. ≥120mmHg; *P* for interaction =0.044) and new-onset NAFLD. The effect of AIP on the risk of NAFLD was smaller in ≥120mmHg group [HR =1.53, 95%CI (1.44, 1.63), *P <*0.001] than in <120mmHg group [HR =1.71, 95%CI (1.56, 1.86), *P* < 0.001]. The interaction of other subgroups and AIP had no significant effect on the risk of NAFLD, and the P values of interaction were all >0.05. (**Table 5**)

**Table 5:** Subgroup analysis for the risk of new-onset NAFLD by baseline AIP quartiles (per SD).

Subgroup	AIP Quartiles (per SD)		
	HR, 95%CI	P value	P for interaction
Sex			0.995
Male	1.59 (1.47, 1.71)	<0.001	
Female	1.59 (1.48, 1.70)	<0.001	
Age group			0.1684
<40	1.65 (1.53, 1.78)	<0.001	
≥40	1.54 (1.44, 1.65)	<0.001	
BMI			<0.001
<20.4	2.32 (1.86, 2.90)	<0.001	
≥20.4 to ≤22.5	1.84 (1.66, 2.03)	<0.001	
≥22.5	1.48 (1.40, 1.57)	<0.001	
ALT			0.103
<16	1.66 (1.50, 1.84)	<0.001	
≥16	1.51 (1.42, 1.60)	<0.001	
SBP			0.044
<120	1.71 (1.56, 1.86)	<0.001	
≥120	1.53 (1.44, 1.63)	<0.001	

Model was adjusted for all covariates in Model 3 except stratifications itself.

### The relationship between AIP and new-onset NAFLD in different BMI groups

More in depth, we analyzed the association between AIP and new-onset NAFLD in different BMI groups. As shown in **Table 6**, compared with the middle and high BMI groups, the association between AIP and new-onset NAFLD was strongest in the low BMI group. In low BMI group, the adjusted HRs of the new-onset NAFLD for subjects in Q4 of AIP was 12.59(4.69, 33.84), significantly higher than middle BMI group [HR =5.42, 95%CI (3.55,8.26)] and high BMI group [HR =3.43, 95%CI (2.65,4.43)].

**Table 6:** The relationship between AIP and new-onset NAFLD in different BMI groups.

AIP Quartiles (per SD)	Q1	Q2	Q3	Q4
Low BMI	Ref.	3.69 (1.32, 10.30) P=0.013	6.66 (2.47, 17.97) P<0.001	12.59 (4.69, 33.84) P<0.001
Middle BMI	Ref.	2.10 (1.33, 3.32) P<0.001	2.52 (1.62, 3.90) P<0.001	5.42 (3.55, 8.26) P<0.001
High BMI	Ref.	1.70 (1.28, 2.26) P<0.001	2.14 (1.64, 2.79) P<0.001	3.43 (2.65, 4.43) P<0.001

Model was adjusted for all covariates in Model 3 except BMI itself.

## Discussion

In this study, we pointed out that AIP was an independent risk factor for new-onset NAFLD in non-obese population. Moreover, AIP was positively associated with the occurrence of NAFLD in non-obese individuals after adjusting for other covariates. It was important to note that this association was significantly different across the BMI subgroups, with the effect strongest in the lowest BMI tertile. In the non-obese population, especially in the low BMI group, the prevention effect of AIP on NAFLD should be emphasized.

With the ongoing awareness of NAFLD disease, non-obese NAFLD was not rare and its prevalence was reported to be up to 12.6% in Korea adults (13). Interestingly, approximately 40% of the global NAFLD population was classified as non-obese NAFLD (14). But since most of the NAFLD with non-obese patients were asymptomatic and prone to the possibility of underdiagnosis, their prevalence may be higher than actual (15). In addition, compared to the obese population with NAFLD, the non-obese population with NAFLD had a higher risk of prostate hyperplasia (16), diabetes mellitus (17, 18), and also had a similar risk of CVD and malignancy (19). However, for the non-obese population, the incidence and severity of dyslipidemia were lower than those of the obese population. Therefore, it was essential to be aware of the non-obese populations with NAFLD and to find appropriate predictors.

AIP, as the logarithm of the TG/HDL-C ratio, combines lipid abnormalities into a lipid complex that can provide a stronger reflection of dyslipidemia and atherosclerosis (5). Currently, the relationship between AIP and NAFLD was not adequately well-studied and limited. To our knowledge, there were only three cross sectional studies on the relationship between AIP and NAFLD, one study concluded that the sensitivity and specificity of AIP(cut-off point 0.045) in predicting NAFLD in Chinese population values 80.8% and 65.4% (6) and may have higher diagnostic capability for women, but still weaker than BMI, which was similar to the results of a study in Chinese Han population (7). Whereas, in another study of NAFLD of obese population, AIP was found to have stronger predictive abilities compared to other indicators, and better in men (8).While in our study, we did not identify the strengths and weaknesses between male and female. The type of study and the size of the sample may contribute to the difference. Nevertheless, in the consideration of the advantages of long-term follow-up retrospective cohort and the large number participants in our study, the credibility of our conclusion was stronger.

Nowadays, a consistent line of research has identified sex difference as a key feature in NAFLD arena (20–22). In addition, menopausal status was also considered as a factor in the development of NAFLD. Thus, we also analyzed the relationship between AIP and NAFLD according to sex and different age groups in female patients. And age 51 years was used as a surrogate for menopause in female patients (21). Our results showed that AIP was also positively and strongly associated with new-onset NAFLD in different sex groups and all different age groups in female patients. In addition, the interaction of sex and AIP had no significant effect on the risk of NAFLD (*P* for interaction >0.05). Although there was no statistical difference (probably limited to sample size), the effect of AIP on the risk of NAFLD showed a trend of weaker predictive capabilities in postmenopausal female patients than premenopausal female patients. This trend may be explained by the fact that estrogen as a protective factor influenced the predictive ability of AIP (22).

The mechanisms of NAFLD pathogenesis were extremely complicated and there were no specific drugs available to treat it. The differences of bile acid metabolism, gut microbiome component (23), genetic predisposition (24) and susceptibility to environmental factors (25) may contribute to the pathogenesis of non-obese NAFLD. In brief, the imbalance between the production of triglycerides(TG) and its uptake and clearance in the liver caused steatosis, which was the key to the occurrence and development of NAFLD (4).

In subgroup analysis, we found a stronger relationship between AIP and new-onset NAFLD in low BMI compared to high and middle BMI, with significant interaction. More intuitively, we observed that association between AIP and new-onset NAFLD was stronger in participants with low BMI than in those with middle BMI and high BMI. We further investigated the distribution of AIP levels in different BMI groups, and the results are shown in **Supplementary Figure 1**. We found that AIP increased with the increase of BMI, so this interaction difference was not related to the increase of AIP in people with low BMI, but the lower AIP in people with low BMI. On the one hand, this effect may be due to the fact that a significant proportion of people in low BMI group were undernutrition. Admittedly, it has been proven to be detrimental to liver no matter people suffering the insufficient or the excessive nutritional status (26). It has been suggested that early nutritional deficiencies lead to abnormal glucose metabolism (27), peroxisomal and mitochondrial dysfunction (28). Combined with such early pathophysiological changes, the clinical manifestations of abnormal lipid metabolism may increase the risk of NAFLD. On the other hand, in developed countries, anorexia nervosa was a common form of nutritional deficiency, which can cause liver damage through hunger-induced autophagy hypothesis (26), thus increasing the incidence of NAFLD in this population Together, these two factors may be responsible for the higher risk of NAFLD in people with elevated AIP (abnormal lipid metabolism) in the low BMI group. In addition, we also found an interaction between AIP and SBP categories on the risk of NAFLD. The association between AIP and new-onset NAFLD was more significant in individuals with SBP < 120mmHg than in individuals with SBP ≥120mmHg. This may be explained by the fact that when individuals with SBP≥120mmHg may have abnormal in other metabolic indicators, which in turn led to a diminished role of AIP.

This study not merely had a large sample size, but also had the advantages of long-term follow-up, retrospective design, and population specificity. Moreover, we used rigorous statistical adjustment to reduce confounding factors to draw more stable conclusions. However, the study still had some limitations. Firstly, our population was only the Chinese non-obese NAFLD population, and there were no data on other ethnic and regional non-obese NAFLD. Secondly, information on smoking, alcohol consumption, waist circumference, hip circumference, etc. was missing from the data, and these may affect the study conclusion. Therefore, a larger population and more comprehensive information are needed to further validate the relationship between AIP and NAFLD in non-obese populations.

## Conclusion

In conclusion, this study found elevated AIP was independently and positively associated with the risk of NAFLD among non-obese patients. Notably, in the low BMI participants, the association between AIP and new-onset NAFLD was more intensive. And the association was screening for AIP could be considered in non-obese people to prevent the new onset NAFLD in the future, and more studies were needed to further confirm the association between AIP and NAFLD in non-obese populations.

## Data availability statement

Publicly available datasets were analyzed in this study. This data can be found here: Dryad data repository at http://datadryad.org/.

## Ethics statement

The studies involving human participants were reviewed and approved by ethics committee of Wenzhou People’s Hospital. The patients/participants provided their written informed consent to participate in this study.

## Author contributions

KL and JNL completed the writing of the paper. JL and XC applied for the database and made statistical analysis. JW was responsible for the revision of the paper. All authors confirmed the final version of the paper.

## Funding

This work was funded by the National Natural Science Foundation of China (Grant No. 81370559 and 81370500 to JNL) and CAMS Initiative for Innovative Medicine (CAMS-2021-I2M-C&T-A-001 to JNL).

## Conflict of interest

The authors declare that the research was conducted in the absence of any commercial or financial relationships that could be construed as a potential conflict of interest.

## Publisher’s note

All claims expressed in this article are solely those of the authors and do not necessarily represent those of their affiliated organizations, or those of the publisher, the editors and the reviewers. Any product that may be evaluated in this article, or claim that may be made by its manufacturer, is not guaranteed or endorsed by the publisher.
