# Supplementary material for: Association between the atherogenic index of plasma and new-onset non-alcoholic fatty liver disease in non-obese participants
Source: Front Endocrinol (Lausanne). 2022 Aug 18;13:969783. doi: 10.3389/fendo.2022.969783 (PMC9433643; doi:10.3389/fendo.2022.969783)
Supplement: Supplementary file 1 [file Image_1.pdf]

Supplemental Figure 1. The distribution of AIP levels in different BMI groups.

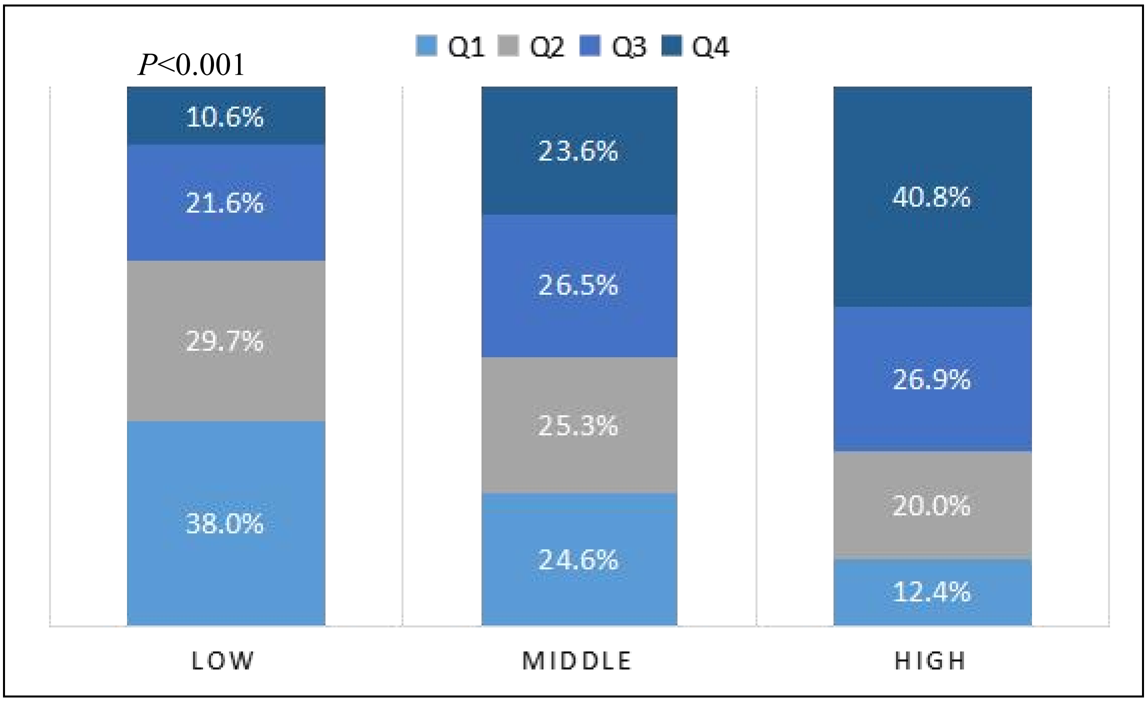

LOW: BMI <20.4 ; MIDDLE: BMI >=20.4 to ＜=22.5 ;HIGH: BMI＞=22.5
