# Supplementary material for: Association between the atherogenic index of plasma and new-onset non-alcoholic fatty liver disease in non-obese participants
Source: Front Endocrinol (Lausanne). 2022 Aug 18;13:969783. doi: 10.3389/fendo.2022.969783 (PMC9433643; doi:10.3389/fendo.2022.969783)
Supplement: Supplementary file 2 [file Table_1.pdf]

Supplemental Table 1. Association between TG, HDL-C and new-onset NAFLD

	Model 1	Model 2<br>HR (95%CI) P value	Model 3
Serum Lipid<br>TG quartiles			
Q1	Ref.	Ref.	Ref.
Q2	2.71 (2. 19, 3.34) P<0.001	2.07 (1.68, 2.56) P<0.001	1.79 (1.42, 2.25) P<0.001
Q3	4.81 (3.95, 5.85) P<0.001	3.06 (2.51, 3.72) P<0.001	2.47 (1.99, 3.06) P<0.001
Q4	11.87 (9.85, 14.30) P<0.001	5.66 (4.69, 6.84) P<0.001	4. 14 (3.36, 5. 10) P<0.001
P for trend (1 Q increment)	2.22 (2. 12, 2.32) <0.0001	1.73 (1.65, 1.82) <0.0001	1.58 (1.50, 1.66) <0.0001
Serum Lipid<br>HDL-C quartiles			
Q1	Ref.	Ref.	Ref.
Q2	0.76 (0.69, 0.84) P<0.001	0.89 (0.81, 0.98) P=0.024	0.90 (0.81, 0.99) P=0.033
Q3	0.49 (0.44, 0.55) P<0.001	0.65 (0.58, 0.73) P<0.001	0.64 (0.56, 0.72) P<0.001
Q4	0.28 (0.24, 0.32) P<0.001	0.42 (0.37, 0.49) P<0.001	0.43 (0.37, 0.50) P<0.001
P for trend (1 Q increment)	0.67 (0.64, 0.69) <0.0001	0.77 (0.74, 0.80) <0.0001	0.77 (0.74, 0.81) <0.0001

Model 1 was not adjusted. Model 2 was adjusted for age, sex and BMI. Model 3 was adjusted for BMI, SBP, DBP, ALP, GGT, ALT, AST, CR, UA.
